# Working memory intervention programs for adults: A systematic review

**DOI:** 10.1590/S1980-57642010DN40300011

**Published:** 2010

**Authors:** Tânia Maria Netto, Denise Vieira Greca, Nicolle Zimmermann, Camila Oliveira, Rochele Paz Fonseca, J. Landeira-Fernandez

**Affiliations:** 1 Ph.D, Psychology Faculty, Post-Graduate Program in Clinical Psychology, Neuroscience, Pontifícia Universidade Católica do Rio de Janeiro (PUC-Rio); Member of the Laboratory “Núcleo de Neuropsicologia Clínica e Experimental” (NNCE), Rio de Janeiro RJ, Brazil.; 2 Member of the Laboratory NNCE, PUC-Rio.; 3 Undergraduate Student, Scholarship Holder PIBIC-CNPq, Psychology Course, Universidade do Vale do Rio dos Sinos, Pontifícia Universidade Católica do Rio Grande do Sul (PUCRS); Member of the Research Group “Neuropsicologia Clínica e Experimental” (GNCE-PUCRS), Porto Alegre RS, Brazil.; 4 Masters Student, Psychology Faculty, Post-Graduate Program in Psychology, Human Cognition Area, PUCRS; Member of the GNCE, Porto Alegre RS, Brazil.; 5 Ph.D, Psychology Faculty, Post-Graduate Program in Psychology, Human Cognition Area, PUCRS; Coordinator of the GNCE; Post-Doctoral Fellow at Centre de Recherche de l’Institute Universitaire de Gériatrie de Montréal (CRIUGM), Université de Montréal, Québec, Canada (CNPq process number 200787/2010-1).; 6 Ph.D, Psychology Faculty, Post-Graduate Program in Clinical Psychology, Neuroscience, PUC-Rio; Coordinator of the Laboratory NNCE. Professor at UNESA, Rio de Janeiro RJ, Brazil.

**Keywords:** working memory, rehabilitation, intervention studies, effectiveness, adults

## Abstract

This systematic review aimed to identify the designs, procedures, and results of empirical studies that performed neuropsychological interventions on WM in adults.

**Methods:**

A PubMed and LILACS literature search was conducted using the keywords *working memory* AND (*training OR rehabilitation OR intervention*) AND *adult*.

**Results:**

Of the seven studies found, three were randomized controlled trials, two were case reports, one was a clinical trial, and one was an evaluation study. With regard to the type of programs and samples, three studies employed global programs with healthy elderly adults and four employed specific programs for samples with neurologically-impaired adults.

**Conclusions:**

The effectiveness of the WM intervention programs was more evident in studies that employed specific methods of rehabilitation for samples with neurological disorders than in those based on global programs with healthy adults. There is a need for more empirical studies to verify the effectiveness of WM intervention programs in order to provide adequate guidance for clinical neuropsychologists and future research.

## Abstract (pt)

Essa revisão sistemática teve como objetivo identificar os delineamentos, procedimentos, e resultados dos estudos empíricos que investigaram intervenções neuropsicológicas de memória de trabalho em adultos.

**Métodos:**

Foi realizada uma busca da literatura nas bases de dados PubMed e LILACS, utilizando as palavras-chave memória de trabalho *working memory* AND (*training OR rehabilitation OR intervention*) AND *adult*, ou memória de trabalho E (treino OU reabilitação OU intervenção) E adulto.

**Resultados:**

De sete estudos encontrados, três eram estudos randomizados controlados, dois eram relatos de casos, um era um ensaio clínico, e um estudo, de avaliação neuropsicológica. Em relação ao tipo de programa e amostra, três estudos foram conduzidos utilizando programas globais de intervenção com idosos saudáveis e quatro utilizaram-se de programas específicos de intervenção com amostras clínicas de pacientes neurológicos.

**Conclusões:**

A efetividade dos programas de intervenção em memória de trabalho foi mais evidente em estudos que empregaram métodos específicos de reabilitação com amostras clínicas neurológicas, quando comparados àqueles baseados em programas gerais de intervenção com adultos saudáveis. Existe necessidade de estudos empíricos que verifiquem a eficácia de programas de intervenção em memória de trabalho que possam contribuir com orientações adequadas para neuropsicológos clínicos e para futuras pesquisas.

This study entailed a systematical review of the evidence-based practices of working memory (WM) intervention programs in the context of clinical neuropsychology and neuroscience. Working memory is a neuropsychological construct, that when impaired, can interfere in simple and complex everyday tasks of an individual’s life. Several studies on WM assessments^1,2^ as well as multiple-memory-system processing^3-5^ can be found in the literature which employ methodological rigor. However, to the best of our knowledge, studies about WM intervention processes with methodological rigor have yet to be fully explored.

Neuropsychological rehabilitation, one of the most studied and well known areas of intervention in neuropsychology, is concerned with the improvement of cognitive, behavioral, emotional, and psychosocial deficits that result from brain injury. Furthermore, it is a process in which damaged individuals work together with a professional team to remedy or alleviate acquired cognitive impairments.^6,7^ With regard to different types of cognitive rehabilitation, including all of its possible features and related variables, no consensual taxonomy can be found in the literature. Considering the number of cognitive domains to be improved, neuropsychological rehabilitation can be divided into two main groups: global and specific. Global rehabilitation seeks to ameliorate several cognitive domains, such as memory, attention, and executive function, whereas specific rehabilitation seeks to improve a particular cognitive function, such as memory.^8^

Working memory is fundamental to the individual processing of complex cognitive thoughts, such as problem solving, language, decision-making, and the execution of actions. It is a multi-component system used not only for temporary storage under attentional control, but also for the manipulation of information. Regarding WM processing, different theoretical models of WM have been proposed in the context of neuroscience. The most influential of these models was proposed by Baddeley and Hitch (1974).^3,9^ In their model, WM consists of three subsystems: the central executive and two others subsidiary slave systems (i.e. the phonological loop and visuospatial sketchpad). The central executive, an attentional controller, is the most important subsystem of the WM multi-component model, coordinating and scheduling mental operations, processing the capacity to focus, divide and switch attention, and provides a link between the two slave systems and long-term memory.^9,10^ According to recent studies, this component appears to have two forms of control. One is automatic, such as consolidated habits (e.g. riding a bike) that require almost no attention, and another which depends on the limited attention of the central executive.^9,11^

In relation to the slave subcomponents, the phonological loop is responsible for the temporary storage of verbal-acoustic information. This system comprises two subcomponents:

[1] the phonological store, in which representations of verbal material, such as word lists, are kept and
[2] a subvocal rehearsal mechanism that maintains information in the phonological loop.^5^

The visuospatial sketchpad is responsible for processing visual and spatial information and consists of two components:

[1] “passive” visual storage and
[2] an active mechanism that maintains the contents of visuospatial storage.

The current model of WM, originally proposed by Baddeley (2000),^4^ has undergone two important changes:

[1] connection of the phonological loop and visual-spatial sketchpad to long-term memory and
[2] the addition of a fourth component, the episodic buffer, which was assumed to have a limited capacity and to directly obtain information from the other WM subcomponents and long-term memory, transforming it into coherent episodes.^11^

The episodic buffer can be defined as an interface between a number of other different cognitive sources, such as visual, verbal, and perceptual codes, and long-term, semantic, and episodic memories. The episodic buffer differs from long-term episodic memory, because of its temporary storage. Also, it has been hypothesized to act as a workplace in conscious awareness.^4,12^

Different neurological and psychiatric pathologies may alter WM, resulting in substantial impairments affecting an individual’s life. Degenerative disease studies have found that WM is affected during the early stages of Alzheimer’s disease^13-16^ and also multiple sclerosis.^17,18^ Moreover, deficits in WM are quite common in brain injury and stroke.^19-23^ Furthermore, neuropsychological deficits in affective disorders have been a topic of increasing research. Initially, research was focused mainly on depression, which found psychomotor slowing and deficits in attention, verbal memory, WM, and executive function.^24,25^ But with time, research has expanded to encompass other affective disorders, including schizophrenia and bipolar disorder. With regard to schizophrenia, deficits in WM and executive function are frequently observed.^26-29^ Similarly, evidence is beginning to emerge that WM may also be a core feature of bipolar disorder.^26^

Based on the relevance of theoretical and methodological issues, the consequences of WM impairments on an individual’s life, and the sparse evidence-based studies on WM intervention programs available in the scientific literature, systematic reviews in this area have become increasingly important. To our knowledge, no systematic reviews have yet been published on WM intervention programs. Only one non-systematic review about training of the executive component of WM was found in the PubMed database.^30^ This previous article investigated different issues to those focused in the present paper and explored the neural basis, transfer effects, and age-related changes after training.

The aim of the present systematic review was to present a spectrum of empirical studies on WM interventions in adults, describing and analyzing their designs, procedures, and results. This knowledge may contribute to evidence-based guidance for clinical practice and future research. In order to achieve these goals, this review attempted to answer the following specific questions:

[1] Which evidence-based studies in the national and international literature have investigated neuropsychological interventions to improve WM in adults?
[2] Amongst the studies selected for this review, what are their main methodological features of designs, samples (control and clinical), assessments, intervention procedures, and theoretical framework?
[3] What are the main results?
[4] Which studies presented clear evidence of neuropsychological intervention effectiveness?

The hypotheses for each research question are the following:

[1] Few studies in the national and international literature have investigated neuropsychological interventions to improve WM in adults.
[2] The methodological features will consist mostly of randomized controlled trials and single-case studies with healthy and clinical samples and WM assessments and intervention procedures based on a theoretical framework.
[3] Working memory training can improve WM performance in neuropsychological tests.
[4] Considering the global and specific types of interventions, the evidence of neuropsychological intervention effectiveness will be attributable to specific approaches.

Additionally, with regard to study design, randomized controlled trials are hypothesized to present the most informative findings.

## Methods

Two databases were consulted: LILACS (Latin American and Caribbean Health Sciences Literature) for national studies, and PubMed for international studies. The terms used were the following: *working memory* AND (*intervention OR rehabilitation OR training*). The abstracts were selected according to the following inclusion criteria:

[1] empirical designs,
[2] English, French, Spanish, or Portuguese written languages,
[3] publication date between 2000-2010,
[4] WM neuropsychological interventions,
[5] WM stimulation procedures clearly specified,
[6] pre and post-intervention assessments and
[7] sample with adult participants.

All studies were selected by one author and three external judges, experts in this area.

The selected variables observed for later analysis were the following:

[1] Objective;
[2] Method: samples (n, age, education, clinical characteristics, inclusion and exclusion criteria), neuropsychological assessment (functions investigated and assessments tools), intervention (design, type of rehabilitation, theoretical framework, procedures and duration time); and
[3] Results.

## Results

The initial searches on the two data bases resulted in a total of 643 articles. More specifically, 40 studies were found on the LILACS data base, and 603 on PubMed. After the post-inclusion criteria analysis, none of the articles from LILACS were included and only 9 from PubMed were selected. These results showed that after the analyses of the inclusion criteria, no national studies were included.

Among the nine abstracts selected after the first screening, two were excluded, one was a study that presented a case report in which the original version was already included in this review, while the other presented WM in its title but had no explicit neuropsychological assessment procedure or WM intervention program. After these exclusions, only 7 studies from PubMed remained, corresponding to 1.08% of the initial search. The main features that did not meet the inclusion criteria among the excluded studies, were pharmacological and non-empirical cognitive interventions, music stimulation, and samples composed of children and adolescents.

Regarding study designs, three articles from the seven selected studies were randomized controlled trials, one a clinical trial, and another an evaluation study, with the last two being case reports. These studies were organized into two types of interventions:

[1] global and
[2] specific.

Two studies belonged to the global rehabilitation category, while five were specific. The majority of the intervention studies used a specific approach and were based on a randomized controlled trial design, whereas case reports were the second most frequent study type.

Table 1 describes the studies based on global intervention categories whereas Table 2, shows those based on specific approaches. In both tables, the main features presented by each study are summarized according to objective, sample, neuropsychological assessment, intervention and results. The results of this analysis are presented in two subsections below: global rehabilitation studies and specific rehabilitation studies.

**Table 1:** Description of the variables analyzed in the global intervention studies.

Reference Objective	Method			Results
	**Sample**	**Neuropsychological assessment**	**Intervention**	
Study ^39^ (Vallat et al., 2005)	(a) Type and age: Healthy older adults, age > 65 years. (b) Groups: Experimental group 1 (n=85), experimental group 2 (n=68), control group (n=85); no sample loss reported. (c) Inclusion criteria: no dementia or memory impairment, adequate intellectual functioning. (d) Exclusion criteria: degenerative neurological disorder; severe psychotic traits, depression, agitation, or behavioral problems; history of alcohol or substance abuse; systemic disease.	(a) Functions and abilities: attention; working memory; immediate execution, logic, recent word list, and short-term memories; learning potential; naming, repetition, auditory, written, and reading language; visuo-constructive ability; planning; bimanual coordination; visuo-manual coordination speed; phonetic and semantic fluency; abstraction; categorization. (b) Frequency: Six assessments of 6 months each - four with the same neuropsychological battery and two with ADAS-COG.	(a) Design: randomized clinical trial; double-blind; longitudinal; quasi-experimental. (b) Type of rehabilitation: group modality; involving all cognitive functions; experimental group 1 (training: cognitive, social skills, alternative therapies, musical therapy, culture); experimental group 2 (similar to group 1, but not following an organized timetable); control group (untrained). (c) Theoretical framework: Developed by the author based on Braak and Braak’s model of Alzheimer’s staging. (d) Procedures: cognitive training of attention and orientation, memory, language, visuo constructive ability, executive function, visuo-manual coordination, and praxia. (e) Duration: 2 years, 180 sessions, 1.5 h per session twice a week.	• Significant improvements in immediate memory in experimental group 1, particularly in the second year. • Recent logic execution memory was significantly improved in all three groups. • Working memory was only statistically significant in experimental group 1 on the second year.
To assess the efficacy and specificity of WM rehabilitation, focusing mainly on central executive and phonological loop.				
Study ^23^ (Westerberg et al., 2007)	(a) Type and age: Healthy independent-living elderly adults, with ages ranging from 71 to 87 years. (b) Groups: Experimental group: early training (n=29); control group (late training, n=20). (c) Inclusion criterion: subjective complaints of cognitive or memory dysfunction.	(a) Functions and abilities: working memory, primary and secondary memory. (b) Frequency: Pre- and post-intervention assessments separated by 3 months each with a 6 month follow-up after training; four different but equivalent batteries.	(a) Design: clinical trial. (b) Type of rehabilitation: group modality, 3 modules: [i] memory skills, [ii] goal management, and [iii] psychosocial training. Both groups were submitted to the same training but at different times. (c) Theoretical framework: Jacoby (1991); difference between two major components of recall: one more automatic and familiarity-based and the other more controlled and recollective. (d) Procedures: memory skills learning and organization, external and internal techniques. (e) Duration: 12 weeks, one session per week.	• No training-related improvement in working, primary, or recognition memory. • Positive results were found only in the experimental group.
To examine the effects of WM training in adult patients with stroke.				

**Table 2:** Description of the variables analyzed in the specific intervention studies.

Reference Objective	Method			Results
	**Sample**	**Neuropsychological assessment**	**Intervention**	
Study ^33^ (Buschkuehl et al., 2008)	(a) Type and age: Healthy older adults, mean age of 80.1. (b) Groups: Experimental group (WM training, n=13), control group (physical training, n=19). (c) Inclusion criteria: absence of acute heart, psychiatric, or debilitating problems; arthrosis problems; independent and healthy elderly adults.	(a) Functions and abilities: WM, episodic memory. (b) Frequency: Pre- and post-intervention, with 1 year follow-up; the same battery was administered.	(a) Design: single-case with multiple-baseline-across behavior with a control group. (b) Type of rehabilitation: training of WM storage and processing components; specific cognitive retraining of the central executive and phonological loop. (c) Theoretical framework: WM model (Baddeley, 1986, 1998). (d) Procedures: Eight different tasks; [i] reconstruction of words from oral spelling, [ii] reconstruction of words from oral spelling with a letter omitted, [iii] oral spelling, [iv] odd or even number of letters in a word, [v] reconstitution of words from syllables, [vi] alphabetizing, [vii] word sorting in alphabetical order, [viii] acronyms. (e) Duration: 6 months, 1 h training sessions three days per week	• Experimental group showed overall increased visual WM performance and, to a lesser degree, visual episodic memory performance. • No differences between groups in the 1 year follow-up.
To investigate the effect of WM training on WM and episodic memory performance				
Study ^34^ (Duval et al., 2008)	(a) Type and age: A case of a 23-year-old, right-handed student, bilingual (French) at an academy of music. (b) Deficits: WM impairment as a result of cerebral tumor surgery on his left temporal lobe.	a) Functions and abilities: memory, language, constructional praxis, intellectual abilities, attention, executive functions. (b) Frequency: Four assessments with the same tests: pre-evaluation, intermediate, post-immediate, and post 3 months.	a) Design: case report, multiple baselines. (b) Type of rehabilitation: cognitive program (training of three WM subcomponents; central executive) complemented by an ecological approach. (c) Theoretical framework: non-passive storage by slave systems (Emerson and Miyake, 2003), complemented by an ecological approach (WM model; Baddeley, 1986). d) Procedures: [i] cognitive rehabilitation (exercises divided into three subprograms: central executive, visual sketchpad, and phonological loop), [ii] ecological rehabilitation (analyses of scenarios and simulations of real-life situations). (e) Duration: 6 months, 90 min sessions four times per week.	• Effectiveness for all three WM components. • Generalization to everyday life, and effects were maintained after 3 months.
To describe and evaluate a program of neuropsychological rehabilitation.				
Study ^20^ (Serino et al., 2007)	(a) Type and age: Traumatic brain injury patients with severe WM deficits, with ages ranging from 16 to 57 years. (b) Group: Experimental group, n=9. (c) Inclusion criteria:≥6 months post-injury; no other neurological disease, no emotional or psychiatric disturbances or communication problems	(a) Functions and abilities: processing speed, sustained and divided attention, WM, long-term memory, executive functions, psychosocial abilities, everyday functioning. (b) Frequency: Three assessments with different versions of the same instruments. One at admission, another after General Stimulation Training and the last at the end of WM training.	(a) Design: pilot study with pre and post-intervention assessment. (b) Type of rehabilitation: General Stimulation Training (low executive demand) followed by WM training. (c) Theoretical framework: WM model (Baddeley, 1986, 2003). (d) Procedures: Three WM tasks; [i] repeated applications of the Paced Auditory Serial Addition Test (PASAT, central executive), and two other tasks from PASAT; [ii] months task ; and [iii] words task. (e) Duration: 2 months (1 month for each intervention phase), four sessions per week.	• WM training was effective in recovering central executive impairments. • Some cognitive functions dependent on the central executive improved. • Everyday life functioning improved. • Significant improvement in WM, divided attention, executive functions, and long-term memory, but not in processing speed or sustained attention.
To investigate the efficacy of a rehabilitation program (WM training) on WM and other cognitive functions dependent on this component system, such as divided attention, executive functions, and long-term memory; to verify whether the improvement generalizes to everyday activities.				
Study ^39^ (Vallat et al., 2005)	(a) Type and age: A case of a 53-year-old right-handed male high school graduate computer scientist.Control group: (n=10) that matched the case's age and education background; this control group performed all therapy tasks with a ceiling effect. (b) Deficits: aphasia and WM central executive and phonological loop impairment as a result of a stroke; complaints of difficulties in everyday tasks.	(a) Functions and abilities: oral language, attention, verbal and visual long-term memory. (b) Frequency: Two pre- and one post-intervention assessment sessions, with similar outcome measures and parallel versions of some tests.	(a) Design: single-case with multiple-baseline-across-behavior with a control group. (b) Type of rehabilitation: training of WM storage and processing components; specific cognitive retraining of the central executive and phonological loop. (c) Theoretical framework: WM model (Baddeley, 1986, 1998). (d) Procedures: Eight different tasks; [i] reconstruction of words from oral spelling, [ii] reconstruction of words from oral spelling with a letter omitted, [iii] oral spelling, [iv] odd or even number of letters in a word, [v] reconstitution of words from syllables, [vi] alphabetizing, [vii] word sorting in alphabetical order, [viii] acronyms. (e) Duration: 6 months, 1 h training sessions three days per week	• Case's forward digit span improved significantly compared with matched controls. • Central executive and phonological store components of WM significantly improved after rehabilitation. • Significant decrease in daily difficulties; return to full-time job at same position as before stroke.
To assess the efficacy and specificity of WM rehabilitation, focusing mainly on central executive and phonological loop.				
Study ^23^ (Westerberg et al., 2007)	(a) Type and age: Stroke patients with ages ranging from 34 to 55 years. (b) Groups: Experimental group (trained, n=9), control group (untrained, n=9). (c) Inclusion criteria: time post-onset between 12 and 36 months; access to internet connection at home; self-reported deficits in attention. (d) Exclusion criteria: IQ < 70, motor or perceptual handicap that would prevent use of computer, medication changes during the study period, major depression, known history of alcohol abuse or illicit drugs.	(a) Functions and abilities: WM, attention, reasoning and problem-solving, declarative memory, inhibition, learning. (b) Frequency: Pre- and post-intervention sessions with the same assessment battery.	(a) Design: randomized pilot study. (b) Type of rehabilitation: computerized training on various WM tasks. (c) Theoretical framework: not reported. (d) Procedures: complete training on a computer at home and daily internet report to a server at the hospital. (e) Tasks employed: [i] reproducing a light sequence in a visuo-spatial grid, [ii] indicating numbers in reverse order, [ii] identifying letter positions in a sequence, [iv] identifying a letter sequence in pseudo words, [v] finding mismatched letters, [vi] reproducing a light sequence in a rotated grid, [vii] reproducing a light sequence in a three-dimensional visuo-spatial grid. (f) Duration: 5 weeks, 40 min sessions 5 days per week, 90 trials per day.	• Statistically significant training effects on non-trained tests for WM and attention. • Significant decrease in symptoms of cognitive problems in daily living. • Some evidence that 1 to 3 years after stroke, intensive training can improve an individual's WM and attention performance.
To examine the effects of WM training in adult patients with stroke.				

### Global rehabilitation studies

Table 1 shows that both studies had the same objectives, which was to verify the effectiveness of neuropsychological interventions in healthy elderly adults by employing a group modality. Moreover, these studies had common inclusion criteria (i.e., self-report of memory complaints). However, the studies differed in several respects, such as sample size (study 31 had a much larger sample than study 32), the number of cognitive functions evaluated (study 32 was limited mainly to memory domains), length of time of the program (study 31 lasted twice as long as study 32), and assessment frequency (study 32 included follow-up and used different versions of the same evaluation battery). Furthermore, these investigations also differed with regard to their methodological designs, training of cognitive domains, length of time (which varied from three months in study 2 to 24 months in study 1), frequency of sessions (from once per week in study 32 to twice per week in study 31), and theoretical framework. The results related to WM improvement were restricted to study 31, which provided other cognitive domain stimulations in the experimental group, in addition to memory training.

### Specific rehabilitation studies

Table 2 summarizes the results of specific intervention studies included in this review and outlines the evidence of several aspects of WM-specific interventions. The main objective of these studies was to evaluate the effectiveness of WM intervention approaches. However, some of these went further and examined other cognitive functions, such as language (studies 34 and 39); attention (studies 34, 20, 39, and 23); executive function (studies 34, 20, and 23); psychosocial ability (study 20); and everyday functioning (study 20). With regard to study design, two investigations described single cases (studies 34 and 39), two had experimental and control groups (study 33 had physically trained, and study 23 had untrained), and one had the experimental group as its own control (baseline intra-group comparison in study 20). Most of the studies had samples of adults with WM impairments (studies 34, 20, 39, and 23), with the exception of one study that investigated healthy and independent octogenarians (study 33). All samples of these investigations had a small n, averaging 11 individuals per group (studies 33, 20, and 23), and two of the studies had just one subject based on single cases (studies 34 and 39). To complete the sample features, most of the eligibility criteria varied according to the type of impairment (studies 34, 20, 39, and 23).

Considering the neuropsychological tests, all studies that employed specific intervention assessed the participants during pre- and post-interventions. Two of the studies also had follow-ups (studies 33 and 34), and one provided assessments during the intervention (study 20). Three studies in Table 2 used the same battery to retest individuals (studies 33, 34, and 7), and the other two employed different test versions (studies 20 and 39). All of the rehabilitation programs described by the studies had WM as the principal cognitive domain. However, in some studies, other cognitive domains were also trained (studies 39 and 23). Moreover, the majority of the interventions were executed in a group modality (studies 33, 20, and 23), and two of the studies used computerized training (studies 33 and 23). With regard to the theoretical framework, three investigations used the model proposed by Baddeley and Hitch (1974) or more recent versions (studies 34, 20 and 39).^4^

High variability was found in terms of the total duration of the programs, which ranged from 5 weeks (study 7) to 6 months (studies 34 and 39). Additionally, the frequency of sessions ranged from three times per week (studies 39 and 23) to five times per week (studies 34 and 20), and the session duration ranged from 40 to 90 min.

Considering the results, all studies demonstrated gains from WM training. Furthermore, four investigations presented a generalization effect to everyday life (studies 34, 20, 39, and 23), and one study demonstrated a transfer effect to cognitive domains related to WM (study 23). After 3 months, follow-up assessment still showed maintenance of WM improvement as a result of two interventions (studies 34 and 39).

## Discussion

In spite of the low number of studies on WM intervention programs found in this review, there was great variability regarding types of designs, theoretical frameworks, samples, assessments, interventions, and results. This heterogeneity can limit the ability of the literature to provide clear direction for future clinical studies. Hereafter, discussion is guided by the answers to the questions initially established in this review.

Which evidence-based studies in the national and international literature have investigated neuropsychological interventions to improve WM in adults?

All studies presented in this review were found on the international PubMed database, demonstrating the need for these types of investigations in the Latin-American literature. This result partially supports the hypotheses that few studies would be found in the national and international literature investigating neuropsychological interventions to improve WM in adults. The small number of studies in this area may be related to the fact that both neuropsychological interventions and WM are relatively new constructs in the context of neuroscience, and studies on their interaction are more recent still.

Furthermore, Cicerone et al. (2000) classified studies into three classes of evidence in regard to the method strength: I, II, and III. Class I refers to prospective studies that are robustly designed, such as randomized controlled trials. Other investigations, such as, quasi-randomized studies, can be classified as Class Ia. Class II includes prospective, nonrandomized cohort, and case-control investigations. Class III consists of studies with no control groups, including case studies [for further details, see ^35^, pg. 1598]. According to this classification standard, three studies in the present review were assigned to Class I,^23,31,33^ one assigned to Class II^32^ and three to Class III.^20,39,34^

Amongst the studies selected for this review, what are their main methodological features of designs, samples (control and clinical), assessments, intervention procedures, and theoretical framework?

The results presented in this review confirmed the hypothesis that the methodological features consist mostly of randomized controlled trials and single case studies with healthy and clinical samples, and that the WM assessment and intervention procedures are based on a theoretical WM framework. This hypothesis originated from evidence-based reviews of cognitive interventions. These types of review usually focus on describing studies that are randomized controlled trials and seldom describe studies such as single cases, except when they provide unique results that can be used for clinical and future research guidance.^35-38^ Three studies in the present review applied the randomized controlled trials method, which although a fairly rigorous methodological design, minimize the heterogeneity of samples and effects of unconventional variables. Moreover, two other studies employed a single case design, which confirmed the hypothesis.

With regard to the types of samples, among the seven analyzed studies, only three investigated healthy older adults. The remaining studies examined brain-injured individuals. this result is in accordance with a general trend in the neuropsychological literature that shows a preference for investigating clinical samples as opposed to healthy individuals. From a historical point of view, the majority of the discoveries in neuropsychology have been derived from brain-damaged individuals. The same is also true for the rehabilitation field.^20,23,35.36.39-41^ Regarding cognitive stimulation of healthy samples, other studies in the literature have stated that one of the most frequently investigated groups in this approach, especially in memory training, are healthy elderly adults.^30,38^ However, very few have verified the effectiveness of WM training in older adults.

The present review revealed that one of the major challenges of WM interventions is to obtain large sample sizes. Only one of the presented studies was able to accomplish this goal. Achieving a large sample size poses a challenge, because giving adequate attention to all members of a group may be difficult when subjects differ in their levels of ability to perform tasks, learn, retain and recall information, or process other cognitive domains in addition to mnemonic ones. Even if these groups are homogenized and these problems are minimized, other challenges still exist.

With regard to the assessments, another issue concerns the test-retest effect which is oftentimes attributable to the lack of different versions of recommended neuropsychological tools in the literature. In fact, this effect becomes a notable issue in healthy participants, especially when the tools are administered in more than one assessment during intervention and follow-ups, because these individuals retain an intact cognitive ability to learn, process, retain, and retrieve information. However, having a control group in these studies can minimize this confounder.^30^ Finally, six of the seven studies used a theoretical approach. Of these six, three were based on the model proposed by Baddeley and Hitch (1974) and Baddeley (1986, 2000). According to Wilson (2008), the majority of neuropsychologists who practice or research rehabilitation believe that interventions should be guided by theory.

What are the main results?

The present review found that six of the seven studies reported WM improvements in performance on neuropsychological tasks. However, one of the six investigations reported significant differences only in the WM visuospatial sketchpad.^33^ These results support the hypothesis of the present review, which stated that WM training can improve WM performance on neuropsychological tests. Overall, the few evidence-based studies available in the literature are generally consistent with regard to the effectiveness of WM interventions especially when rehabilitating brain-damaged individuals.^20,23,30,31,33,34,39^

According to the results of this review, it seems that healthy elderly adults require longer intervention times than brain-injured adults, in order to demonstrate improvement in the performance on most WM system measurements. A possible explanation may be that these elderly adults are much closer to the norms of performance in neuropsychological tests than brain-injured adults. However, there was a study selected in this review,^33^ involving octogenarians adults, that had a duration of three months. Despite this short period the participants still showed improvement in test performance, but in only one WM system. Therefore, the cited study shows that even over a short timeframe, at least one WM system can be trained in octogenarian adults.

Which studies presented clear evidence of neuropsychological intervention effectiveness?

Among the seven international studies, six provided evidence supporting the effectiveness of WM-specific training in adults. Furthermore, the study design that presented the most informative findings was the randomized clinical trial.^35,36^ This trial supports our initial hypothesis that the evidence of effectiveness could be related to a specific approach. According to other reviews, studies employing specific WM interventions usually present positive performance in the measurements. Specific cognitive interventions may act differentially on different memory domains, and more specific tasks that stimulate a specific WM component will result in greater improvements.^30,36^ Studies that use a robust methodological design, such as a randomized control trial, indeed show more consistent results. Therefore, valuable systematic reviews are usually based on such studies to guide clinical intervention.^36^

With regard to the effectiveness of WM interventions, two main concerns can be derived from the literature: generalization and the transfer effect. Gains acquired during an intervention and subsequently applied to real-life situations are referred to as generalization. Three studies in the present review were successful in this regard.^23,34,39^ However, one challenge in this area is to maintain these gains. The transfer effect occurs when an untrained task is improved as a result of a trained task. For example, an intervention goal is to train the WM domain and as a result improves not only this domain, but others that were not trained such as language or episodic and semantic memory. Only one study in this review was found to have this effect.^23^ Some authors have reported that not enough studies have demonstrated the transfer effect, in spite of it being one of the most important aims in cognitive interventions.^30^

This review demonstrated that the WM domain can improve, especially in brain-damaged individuals. However, these results need to be taken with caution because of the heterogeneity among investigations in terms of designs, samples, assessments, interventions, and the availability of only a few robust methodological designs.

Additionally, more intervention investigations are needed focusing on a single type of cognitive function (e.g., WM in the present review), because if several cognitive components are stimulated in the same program, determining which ones were successful may be difficult, even when specific measurements are used for each cognitive domain. In real-life situations, this may not be the best solution, because individual’s cognition is complex and may require several treatment approaches to improve different functions in several situations. One solution could be to divide the training program into different modules and investigate one particular cognitive domain in the first module before initiating other subsequent training.

As a final suggestion for future studies in the area of WM intervention programs with adults, it is important to carefully plan the length of the intervention process, the frequency of sessions per week, the amount and types of training tasks,^30^ among other essential methodological factors. These careful steps can help facilitate the replication of such studies.

## References

[1] Busch RM, Chapin JS (2008). Review of normative data for common screening measures used to
evaluate cognitive functioning in elderly individuals. Clin Neuropsychol.

[2] Peña-Casanova J, Quiñones-Ubeda S, Quintana-Aparicio M (2009). Spanish Multicenter Normative Studies (NEURONORMA Project): norms
for verbal span, visuospatial span, letter and number sequencing, trail
making test, and symbol digit modalities test. Arch Clin Neuropsychol.

[3] Baddeley A, Hitch GJ, Bower GA (1974). Working Memory. The psychology of learning and motivation: volume 8. Advances in
Research and Theory.

[4] Baddeley A (2000). The episodic buffer: a new component of working
memory?. Trends Cogn Sci.

[5] Baddeley A, Hitch GJ, Allen RJ (2009). Working memory and binding in sentence recall. J Mem Lang.

[6] Wilson BA (2008). Neuropsychological rehabilitation. Annu Rev Clin Psychol.

[7] Wilson BA (2002). Cognitive rehabilitation in the 21st century. Neurorehabil Neural Repair.

[8] Francés I, Barandiarán M, Marcellán T, Moreno L (2003). Psychocognitive stimulation in dementias. Na Sist Sanit Navar.

[9] Baddeley AD (1996). Exploring the central executive. Q J Exp Psychol A Human Exp Psychol.

[10] Baddeley A, Baddeley AD (2007). Fractionating the central executive. Working memory, thought and action.

[11] Baddeley A, Baddeley AD, Eysenck MW, Anderson MC (2009). Working memory. Memory.

[12] Baddeley A, Baddeley AD (2007). Long term and the episodic buffer. Working memory, thought and action.

[13] Huntley JD, Howard RJ (2010). Working memory in early Alzheimer's disease: a neuropsychological
review. Int J Geriatr Psychiatry.

[14] Lim HK, Juh R, Pae CU, Lee BT, Yoo SS, Ryu SH, Kwak KR, Lee C, Lee CU (2008). Altered verbal working memory process in patients with
Alzheimer's disease: an FMRI investigation. Neuropsychobiology.

[15] MacPherson SE, Della Sala S, Logie RH, Wilcock GK (2007). Specific AD impairment in concurrent performance of two memory
tasks. Cortex.

[16] Sebastian MV, Menor J, Elosua MR (2006). Attentional dysfunction of the central executive in AD: evidence
from dual task and perseveration errors. Cortex.

[17] Chiaravalloti N, Hillary F, Ricker J, Christodoulou C, Kalnin A, Liu WC, Steffener J, DeLuca J (2005). Cerebral activation patterns during working memory performance in
multiple sclerosis using fMRI. J Clin Exp Neuropsychol.

[18] Hildebrandt H, Lanz M, Hahn HK, Hoffmann E, Schwarze B, Schwendemann G, Kraus JA (2007). Cognitive training in MS: effects and relation to brain
atrophy. Restor Neurol Neurosci.

[19] Philipose LE, Alphs H, Prabhakaran V, Hillis AE (2007). Testing conclusions from functional imaging of working memory
with data from acute stroke. Behav Neurol.

[20] Serino A, Ciaramelli E, Santantonio AD, Malagù S, Servadei F, Làdavas E (2007). A pilot study for rehabilitation of central executive deficits
after traumatic brain injury. Brain Inj.

[21] Vallat-Azouvi C, Weber T, Legrand L, Azouvi P (2007). Working memory after severe traumatic brain
injury. J Int Neuropsychol Soc.

[22] Vallat-Azouvi C, Pradat-Diehl P, Azouvi P (2009). Rehabilitation of the central executive of working memory after a
severe traumatic brain injury: two single-case studies. Brain Inj.

[23] Westerberg H, Jacobaeus H, Hirvikoski T, Clevberger P, Ostensson ML, Bartfai A, Klingberg T (2007). Computerized working memory training after stroke: a pilot
study. Brain Inj.

[24] Marquand AF, Mourão-Miranda J, Brammer MJ, Cleare AJ, Fu CH (2008). Neuroanatomy of verbal working memory as a diagnostic biomarker
for depression. Neuroreport.

[25] Mondal S, Sharma VK, Das S, Goswami U, Gandhi A (2007). Neuro-cognitive functions in patients of major
depression. Indian J Physiol Pharmacol.

[26] Green MF (2006). Cognitive impairment and functional outcome in schizophrenia and
bipolar disorder. J Clin Psychiatry.

[27] Sánchez-Morla EM, Barabash A, Martínez-Vizcaíno V, Tabarés-Seisdedos R, Balanzá-Martínez V, Cabranes-Díaz JA, Baca-Baldomero E, Gómez JL (2009). Comparative study of neurocognitive function in euthymic bipolar
patients and stabilized schizophrenic patients. Psychiatry Res.

[28] Pae CU, Juh R, Yoo SS, Choi BG, Lim HK, Lee C, Paik H, Jeun SS, Lee CU (2008). Verbal working memory dysfunction in schizophrenia: an fMRI
investigation. Int J Neurosci.

[29] Pachou E, Vourkas M, Simos P, Smit D, Stam CJ, Tsirka V, Micheloyannis S (2008). Working memory in schizophrenia: an EEG study using power
spectrum and coherence analysis to estimate cortical activation and network
behavior. Brain Topogr.

[30] Dahlin E, Bäckman L, Neely AS, Nyberg L (2009). Training of the executive component of working memory:
subcortical areas mediate transfer effects. Restor Neurol Neurosci.

[31] Buiza C, Etxeberria I, Galdona N, Gonzalez MF, Arriola E, Lopez de Munain A, Urdaneta E, Yanguas JJ (2008). A randomized, two-year study of the efficacy of cognitive
intervention on elderly people: the Donostia Longitudinal
Study. Int J Geriatr Psychiatry.

[32] Craik FIM, Winocur G, Palmer H, Binns MA, Edwards M, Bridges K, Glazer P, Chavannes R, Stuss DT (2007). Cognitive rehabilitation in the elderly: effects on
memory. J Int Neuropsychol Soc.

[33] Buschkuehl M, Jaeggi SM, Hutchison S, Perrig-Chiello P, Dapp C, Muller M, Breil F, Hoppeler H, Perrig WJ (2008). Impact of working memory training on memory performance in
old-old adults. Psychol Aging.

[34] Duval J, Coyette F, Seron X (2008). Rehabilitation of the central executive component of working
memory: a re-organisation approach applied to a single case. Neuropsychol Rehab.

[35] Cicerone KD, Dahlberg C, Kalmar K, Langenbahn DM, Malec JF, Bergquist TF, Felicetti T, Giacino JT, Harley JP, Harrington DE, Herzog J, Kneipp S, Laatsch L, Morse PA (2000). Evidence-based cognitive rehabilitation: recommendations for
clinical practice. Arch Phys Med Rehabil.

[36] Cicerone KD, Dahlberg C, Malec JF, Langenbahn DM, Felicetti T, Kneipp S, Ellmo W, Kalmar K, Giacino JT, Harley JP, Laatsch L, Morse PA, Catanese J (2005). Evidence-based cognitive rehabilitation: updated review of the
literature from 1998 through 2002. Arch Phys Med Rehabil.

[37] Teasell R, Bayona N, Marshall S (2007). A systematic review of the rehabilitation of moderate to severe
acquired brain injuries. Brain Inj.

[38] Zehnder F, Martin M, Altgassen M, Clare L (2009). Memory training effects in old age as markers of plasticity: a
meta-analysis. Restorative Neurol Neuroci.

[39] Vallat C, Azouvi P, Hardisson H, Meffert R, Tessier C, Pradat-Diehl P (2005). Rehabilitation of verbal working memory after left hemisphere
stroke. Brain Inj.

[40] Azouvi P, Couillet J, Leclercq M, Martin Y, Asloun S, Rousseaux M (2004). Divided attention and mental effort after severe traumatic brain
injury. Neuropsychologia.

[41] Cullen N, Chundamala J, Bayley M, Jutai J (2007). The efficacy of acquired brain injury
rehabilitation. Brain Inj.

[42] Jaeggi SM, Buschkuehl M, Jonides J, Perring WJ (2008). Improving fluid intelligence with training on working
memory. Proc Natl Acad Sci U S A.

